# Apparent Temperature and Cause-Specific Emergency Hospital Admissions in Greater Copenhagen, Denmark

**DOI:** 10.1371/journal.pone.0022904

**Published:** 2011-07-29

**Authors:** Janine Wichmann, Zorana Andersen, Matthias Ketzel, Thomas Ellermann, Steffen Loft

**Affiliations:** 1 Section of Environmental Health, University of Copenhagen, Copenhagen, Denmark; 2 Institute of Cancer Epidemiology, Danish Cancer Society, Copenhagen, Denmark; 3 Department of Environmental Science, Aarhus University, Aarhus, Denmark; University of Liverpool, United Kingdom

## Abstract

One of the key climate change factors, temperature, has potentially grave implications for human health. We report the first attempt to investigate the association between the daily 3-hour maximum apparent temperature (Tapp_max_) and respiratory (RD), cardiovascular (CVD), and cerebrovascular (CBD) emergency hospital admissions in Copenhagen, controlling for air pollution. The study period covered 1 January 2002−31 December 2006, stratified in warm and cold periods. A case-crossover design was applied. Susceptibility (effect modification) by age, sex, and socio-economic status was investigated. For an IQR (8°C) increase in the 5-day cumulative average of Tapp_max_, a 7% (95% CI: 1%, 13%) increase in the RD admission rate was observed in the warm period whereas an inverse association was found with CVD (−8%, 95% CI: −13%, −4%), and none with CBD. There was no association between the 5-day cumulative average of Tapp_max_ during the cold period and any of the cause-specific admissions, except in some susceptible groups: a negative association for RD in the oldest age group and a positive association for CVD in men and the second highest SES group. In conclusion, an increase in Tapp_max_ is associated with a slight increase in RD and decrease in CVD admissions during the warmer months.

## Introduction

The influence of weather changes on human health is well known since the era of Hippocrates (430 BC) [1]. The effect of certain weather types (heat waves and air mass types), specific weather parameters, and also of the atmospheric environment in general on human health, particularly all-cause mortality, has been studied extensively [2]–[5]. The Intergovernmental Panel on Climate Change (IPCC) and the World Health Organisation highlight that global climate change will have various impacts, some of which are positive, but mostly negative, on human health [6], [7]. Rising temperature is one of the key climatic change factors with direct effects on health.

Clarifying the relationship between key climate change factors and *specific* health outcomes can assist in identifying vulnerable populations and aid policy makers in formulating preventive actions. Few studies investigated the relationship between *non-heat* wave temperature and cause-specific mortality [2], [3], morbidity [8]–[12], or were conducted in Scandinavia [13]–[16]. In a colder climate the increase of global temperature may benefit health [4], although the wintertime increase in total non-accidental mortality may be due to infectious disease and not direct effect of cold weather [17]. However, few studies investigated the relationship between raised temperature and mortality and morbidity during the colder seasons [4], [18]–[20]. It is likely that the overall effect of raised temperature strongly depends on the cause and type of health outcome (death or hospital admission) and population characteristics (age, sex, socio-economic status (SES)), and the efficiency of the health system. Moreover, air pollution may interact with temperature.

The aim of the study was to investigate the association between the daily 3-hour maximum apparent temperature (Tapp_max_) and total respiratory (RD), cardiovascular (CVD), and cerebrovascular (CBD) emergency hospital admissions between 2002–2006 in Greater Copenhagen, Denmark. Confounding by ambient concentrations of PM_10_ (particulate matter <10 µm in diameter), nitrogen dioxide (NO_2_), and carbon monoxide (CO) was considered along with effect modification by age, sex and SES.

## Methods

### Health outcome, temperature, and confounder definition

Hospital admission data were retrieved from the Danish Hospital Discharge Register for inhabitants of Greater Copenhagen (postal code <2930, ≤15 km radius from the city centre, population ≈1 million) who were >18 years and lived in the area between 1 January 2002−31 December 2006. All hospital admissions with International Classification of Diseases 10th Revision (ICD 10) codes I00−I52 (CVD), J00−J99 (RD) and I60−I69 (CBD) were included. Only primary diagnosed and emergency hospital admissions were included.

Meteorological and air pollution data were measured at the Copenhagen urban background monitoring station by the Department of Environmental Sciences, Aarhus University [21]. The urban background monitoring station is located on the roof of a 20 m high building in the centre of Copenhagen about 300 m east and 50 m west of a street with typical weekday traffic flows of 26 000 and 56 000 vehicles respectively, and minimal contribution from local pollution sources in accordance with WHO guidelines.

Air pollution data included measurements of PM_10_ (Beta attenuation by SM200 monitor; Opsis, Sweden), NO_2_ (M 200A; API, San Diego, USA) and CO (M 300 monitor; API, San Diego, USA). Ozone (O_3_) data were not used due to large number of days with missing measurements. Temperature and relative humidity (RH) were measured with the HMP45a probe (Vaisala, Helsinki). PM_10_, NO_2_ and CO data were applied as 24-hour averages (midnight to midnight). NO_2_ was also used as a daily 1-hour maximum (NO_2max_). During the study period there were 147 and 67 days with missing values for the pollutants and meteorological variables, respectively, with a total of 182 days with missing data out of 1 826 days.

The primary exposure variable was a 3-hour maximum apparent temperature (Tapp_max_). Apparent temperature is a construct intended to reflect the physiological experience of combined exposure to humidity and temperature and thereby better capture the response on health than temperature alone [2], [3]. The measurements of RH have a minor error, which is most likely due to the calibration. However, this has a minor impact on the calculation of Tapp_max_.

Influenza epidemics data were provided by the National Serum Institute as weekly percentage of total general physician's consultations due to influenza in Denmark, whereas city level data were not available.

Addresses of the 70 061 hospitalised persons were retrieved by linkage with the Danish personal identification number registry. A recent report was published on SES groups in Greater Copenhagen, which classified communities and the inner city neighbourhoods into four SES groups, based on household income, educational and employment status [22]. An area SES class was assigned to each person by linking the home street code to a geographical information system dataset. The vast majority (90%) of the 70 061 hospitalised persons lived at only one address during 2002–2006. A SES class could not be assigned to 10 146 hospital admissions (5 094 people) due to invalid street codes. A SES code was assigned for the valid address at which the person lived longest. In the case of more than three addresses, the mode of the area SES classes at the different addresses was assigned to that person.

### Statistical analyses

The time-stratified case-crossover design was applied to investigate the association between Tapp_max_ and the cause-specific hospital admissions. The case-crossover design was developed as a variant of the case-control design to study the effects of transient exposures on emergency events, comparing each person's exposure in a time period just prior to a case-defining event with person's exposure at other times [23]. Hereby, control on all measured and unmeasured personal characteristics that do not vary over a short time period is accomplished. If in addition, the control days are chosen close to the event day, personal characteristics that vary slowly over time are also controlled by matching. A time-stratified approach was applied to select the control days, defining the day of hospital admission as the case day and same day of the week in the same month and year as control days. With this approach even very strong confounding of exposure by seasonal patterns is controlled by design [24]–[27]. The data were analysed using conditional logistic regression analysis (PROC PHREG in SAS 9.2, SAS Institute, Cary, NC).

Public holidays were controlled for by use of dummy variables. A previous study in Copenhagen reported a linear relationship between the air pollutants and the cause-specific admissions for the period 1999–2004 [28]. The pollutants were therefore modelled as linear terms, one pollutant at a time.

Lag0 (same day exposure as day of admission) to lag5 (exposure five days prior to day of admission) of Tapp_max_ were investigated, as well as cumulative averages: mean of lag0-1 (2-day moving average, CA2), and up to mean lag0-4 (CA5) (Figures S1, S2, S2, S4, S5, Text S1). Control days for lag1 to 5 were defined as for lag0. The lag of Tapp_max_ with the lowest Akaike Information Criterion (AIC) was applied in the stratified models. In general, the lowest AIC model had the strongest association (i.e. highest absolute association measure) between Tapp_max_ and a cause-specific outcome. A large European study applied longer lags up to CA15 for the cold period [19]. We did not find any evidence of a delayed effect in the cold period (Figure S6, Text S2).

Hazard ratios (HR) and the 95% confidence intervals (CI) were calculated per inter-quartile range (IQR) increase in Tapp_max_ (in °C). The results are presented as the percent excess risk in cause-specific admissions per IQR increase in Tapp_max_ using the following calculation: β^(HR – 1) × 100%^, where β is the model estimate.

Models were first stratified by seasonal period (warm or cold). Figure S7 indicates the average number of cause-specific hospital admissions per Tapp_max_ (lag0). We did not observe a Tapp_max_ threshold in Copenhagen for which a minimum number of cause-specific hospital admissions occurred (Text S3). We therefore split a year into a warm and cold period. The warm and cold periods were defined as April–September and October–March, respectively, as for Stockholm and cities [8], [10].

Due to the nature of the case-crossover design where each person is his/her own control, susceptibility cannot be investigated by including an interaction term between the susceptibility variable and Tapp_max_. Susceptibility was therefore investigated in stratified analyses by sex, age and SES groups. Age was categorised as 19–65, 66–80 and >80 years.

Sensitivity analyses were applied. The linearity of the relationship between Tapp_max_ and each cause-specific outcome was confirmed in the case-crossover design with the use of restricted cubic spline variables of Tapp_max_ (4 knots), in the *survival* and *design* packages in R statistical software (R Development Core Team, 2010) (results not shown).

Other sensitivity analyses included applying the 24-hour average temperature as an alternative temperature definition, whilst also adjusting for the 24-hour average RH and air pollution levels, public holidays and influenza epidemic (Figures S8 and S9, Tables S4, S5, S6, S7, S8, Text S4). Interaction between Tapp_max_ and PM_10_ was investigated by including an interaction term in the RD and CVD models. The number of hospital admissions in our study provided a 78–98% power to detect a significant association at the 95% level.

## Results

The results are based on 50 096 RD, 60 545 CVD and 17 941 CBD emergency hospital admissions for 23 078, 33 487 and 13 496 people (>18 years), respectively.

Table 1 displays a summary of the meteorological conditions, air pollution levels, and influenza epidemics during the study period. None of the EU air quality limit values were exceeded at the urban background level (PM_10_: 40 µg.m^−3^ (annual), NO_2_: 21 ppb (annual), CO: 5.3 ppm (1-hour max))_,_ but PM_10_ and NO_2_ limit values were exceeded at street level (not shown) [21]. Table 2 displays the Spearman correlations between Tapp_max_ and air pollutants in the warm and cold periods.

**Table 1:** Air pollutant levels and meteorological conditions in Greater Copenhagen and influenza epidemics in Denmark during study period (1 January 2002−31 December 2006).

	All year	Warm period	Cold period
**Number of days**	1 826	915	911
**Tapp_max_ (°C)**			
Number of days with missing data	67	26	41
Mean ± SD	10±8	16±6	4±5
Minimum	−8	0	−8
Maximum	30	30	18
Percentiles			
25^th^	3	12	0
50^th^	10	17	3
75^th^	17	20	7
Inter-quartile range	14	8	7
**PM_10_ (µg/m^3^)**			
Number of days with missing data	37	7	30
Mean ± SD	27±14	27±12	28±15
**NO_2_ (ppb)**			
Number of days with missing data	70	29	41
Mean ± SD	12±5	11±4	13±6
**NO_2max_ (ppb)**			
Number of days with missing data	51	21	30
Mean ± SD	22±10	22±9	23±10
**CO (ppm)**			
Number of days with missing data	113	77	36
Mean ± SD	0.27±0.10	0.22±0.06	0.32±0.10
**Weekly GP visits due to influenza in Denmark (%)**			
Number of days with missing data	0	0	0
Percentiles			
25^th^	0.00	0.00	0.90
50^th^	0.60	0.00	1.10
75^th^	1.30	0.50	2.00
Inter-quartile range	1.30	0.50	1.10

Warm period: April–September, Cold period: October–March.

SD: Standard deviation, GP: General practitioner.

**Table 2:** Spearman correlation coefficient between Tapp_max_ and pollutants in Greater Copenhagen during 1 January 2002−31 December 2006.

Cold period	PM_10_	CO	NO_2_	NO_2max_
**Tapp_max_**	0.051^a^, 854^b^, 0.134^c^	−0.280, 855, <.0001	0.036, 849, 0.291	0.013, 855, 0.709
**PM_10_**	−	0.549, 864, <.0001	0.475, 859, <.0001	0.294, 868, <.0001
**CO**	−	−	0.707, 869, <.0001	0.581, 870, <.0001

Warm period	PM_10_	CO	NO_2_	NO_2max_
**Tapp_max_**	0.341, 884, <.0001	−0.192, 823, <.0001	0.088, 869, 0.009	0.107, 875, 0.002
**PM_10_**	−	0.462, 833, <.0001	0.440, 881, <.0001	0.358, 889, <.0001
**CO**	−	−	0.571, 826, <.0001	0.498, 830, <.0001

Cold period: October–March, Warm period: April–September.

a Spearman correlation coefficient.

b Number of days with data.

c p-value.

The 5-day cumulative average (CA5) of Tapp_max_ and the air pollutants were selected for inclusion in the final models presented here. Results for individual lags and cumulative averages for both temperature and air pollutants, as well as two pollutant models with CA5 of Tapp_max_ and pollutants are presented and discussed in the supplementary material (Figures S1, S2, S3, S4, S5, Tables S1, S2, S3, Text S1). The RD and CVD models were adjusted for PM_10_, whilst the CBD models were adjusted for NO_2max_ in the warm period (Table 3). The RD models were adjusted for PM_10_ in the cold period (Table 4).

**Table 3:** Association between Tapp_max_ and hospital admissions, by cause, expressed as percentage increase in risk (%) and 95% confidence intervals per inter-quartile increase in 5-day cumulative average of Tapp_max_ (in °C) during the warm period of 1 January 2002−31 December 2006 in Greater Copenhagen.

	Respiratory disease^a^ ^b^					Cardiovascular disease^a^ ^b^					Cerebrovascular disease^a^ ^c^				
	IQR	n^d^	%	95% CI		IQR	n	%	95% CI		IQR	n	%	95% CI	
**All**	8	20350	**6.5**	**0.7**	**12.6**	8	25872	**−8.4**	**−12.9**	**−3.7**	8	7762	0.3	−7.0	8.2
**Age categories**															
19–65 years	8	5036	4.4	**−**6.7	16.9	8	8511	**−9.9**	**−17.5**	**−1.7**	8	2032	−5.7	−18.9	9.7
66–80 years	8	8925	**9.8**	**0.9**	**19.5**	8	9497	−1.7	−9.6	6.8	8	2888	−0.7	−12.2	12.4
>80 years	8	6389	3.8	−6.1	14.7	8	7865	**−14.3**	**−21.8**	**−6.1**	8	2842	5.7	−6.8	19.7
**Sex**															
Women	8	11472	**9.5**	**1.6**	**18.0**	8	11740	**−12.2**	**−18.5**	**−5.4**	8	4094	0.8	−9.1	11.8
Men	8	8878	2.8	−5.6	11.9	8	14132	−5.2	−11.4	1.5	8	3668	−0.3	−10.8	11.5
**Socio-economic status**															
Lowest	8	6819	**10.8**	**0.6**	**22.1**	7	7975	**−14.5**	**−21.0**	**−7.4**	8	1811	6.9	−9.0	25.6
Second lowest	8	5287	−1.3	−11.6	10.1	8	6807	−4.4	−13.3	5.5	8	2154	5.3	−8.9	21.7
Second highest	8	4572	**13.2**	**0.5**	**27.5**	8	6053	−5.2	−14.5	5.2	8	2053	−2.7	−15.9	12.6
Highest	8	1909	5.6	−11.8	26.5	8	3065	4.4	−9.8	20.9	8	1273	−0.2	−16.8	19.9

Warm period: April–September.

a Adjusted for public holidays and influenza rates.

b Adjusted for 5-day cumulative average of PM_10_ ((lag0+lag1+lag2+lag3+lag4)/5)

c Adjusted for 5-day cumulative average of NO_2max_

d Number of admissions.

**Table 4:** Association between Tapp_max_ and hospital admissions, by cause, expressed as percentage increase in risk (%) and 95% confidence intervals per inter-quartile increase in 5-day cumulative average of Tapp_max_ (in °C) during the cold period of 1 January 2002−31 December 2006 in Greater Copenhagen.

	Respiratory disease^a^ ^b^					Cardiovascular disease^a^					Cerebrovascular disease^a^				
	IQR	n^c^	%	95% CI		IQR	n	%	95% CI		IQR	n	%	95% CI	
**All**	7	22593	−3.9	−7.6	0.0	7	27911	3.5	−0.1	7.2	7	8064	0.8	−5.6	7.6
**Age categories**															
19–65 years	7	5551	−0.6	−8.2	7.7	7	8988	3.7	−2.5	10.4	7	2056	10.4	−2.9	25.6
66–80 years	7	9789	−0.7	−6.5	5.5	7	10130	3.0	−2.8	9.2	7	3121	0.7	−9.4	11.9
>80 years	7	7253	**−10.6**	**−16.7**	**−4.0**	7	8793	3.6	−2.6	10.4	7	2887	−5.8	−15.7	5.2
**Sex**															
Women	7	12982	−4.6	−9.5	0.5	7	12785	0.2	−4.9	5.5	7	4199	0.3	−8.5	10.0
Men	7	9611	−3.0	−8.8	3.1	7	15126	**6.4**	**1.4**	**11.7**	7	3865	1.3	−7.8	11.3
**Socio-economic status**															
Lowest	7	7605	−1.2	−7.7	5.8	7	8471	3.8	−2.6	10.6	7	1855	15.3	0.7	31.9
Second lowest	7	5563	−3.3	−10.7	4.8	7	7414	1.0	−5.6	8.1	7	2291	−5.0	−16.0	7.5
Second highest	7	5015	−4.2	−12.0	4.3	7	6550	**9.7**	**2.0**	**17.9**	7	2043	5.9	−7.4	21.2
Highest	6	2324	0.0	−10.0	11.1	7	3329	−7.5	−16.5	2.5	7	1293	−5.3	−19.5	11.4

Cold period: October–March.

a Adjusted for public holidays and influenza rates.

b Adjusted for 5-day cumulative average of PM_10_ ((lag0+lag1+lag2+lag3+lag4)/5).

c Number of admissions.

In the warm period, an IQR increase in Tapp_max_ was associated with an increase of 7% (95% CI: 1%, 13%) in RD admissions (Table 3). Stronger associations were observed for the 66–80 year age group, women, lowest and second highest SES groups. For an IQR increase in Tapp_max_ there was a decrease of 8% (95% CI: 4%, 13%) in CVD admissions, strongest for the oldest age group, women, and lowest SES groups. There was no association between Tapp_max_ and CBD admissions in the warm period (Table 3) or with any of the cause-specific admissions in the cold period, except with RD admissions for the oldest age group (negative association) and with CVD admissions for men and the second highest SES groups (positive association) (Table 4).

No interaction between Tapp_max_ and the air pollutants was detected for any of the cause-specific admissions. The robustness of the observed associations was confirmed in models with an alternative temperature definition: the same lag structure and similar effect estimates were observed (Figures S8 and S9, Tables S4, S5, S6, S7, S8, Text S4).

## Discussion

This is the first attempt to evaluate the association between temperature and RD, CVD and CBD emergency hospital admissions in Greater Copenhagen, and this included possible interaction with air pollution and effect modification by sex, age and SES.

We observed a modest increase of 0.8% per 1°C (95% CI: 0.1%, 1.5%) in total RD *emergency* admissions with rising Tapp_max_ in the warm period. Studies from North-Continental cities in Europe, California and London, UK reported similar associations between total RD admissions (emergency or planned) and Tapp_max_, Tapp or T_ave_ (per 1°C) during the warm period [8]–[10]. The Californian study estimated Tapp for each home by a geographical information system model [9]. The British study identified a threshold of 23°C, while the North-Continental study restricted the analyses above the city specific 90^th^ percentile of Tapp_max_ (20–29°C) [8], [10].

An apparent protective effect of high Tapp_max_ on total CVD *emergency* admissions was observed in the warm season: 1.1% decrease per 1°C increase (95% CI: 0.5%, 1.7%), which is in agreement with a weak protective effect on total CVD admissions (emergency or planned) reported for the North-Continental European cities and in the Californian study [8], [9]. A study from London failed to find association between temperature and total CVD admissions (emergency or planned) [10]. A large US study observed a slight increase in total CVD *emergency* admissions with increasing temperature (all year) [11].

Few studies have investigated the association between temperature and total CBD admissions with inconsistent results [8]–[10], [12]. The lack of association between Tapp_max_ and total CBD *emergency* admissions in the warm period in Copenhagen corroborate findings from large studies in Europe and California [8], [9]. Studies from California and the UK reported inverse associations between temperature and total CBD admissions [10], [12].

There was no association with Tapp_max_ during the cold period and any of the cause-specific admissions, except with RD and CVD for some susceptible groups. Studies that focus on temperature effects on hospital admissions in the cold months are lacking.

We did not observe a Tapp_max_ threshold in Copenhagen for which a minimum number of cause-specific hospital admissions occurred (Figure S7, Text S3). However, the weight (number of days) of each Tapp_max_ is different and is taken into account in regression analyses. A linear negative (insignificant) and positive relationship between RD hospital admissions, and Tapp_max_ was observed during the cold and warm periods, respectively. For CVD hospital admissions a positive (insignificant) and negative relationship was observed during the cold and warm periods, respectively. However, these weak associations are related to the absolute Tapp_max_, whereas our case-crossover study focuses on short-term Tapp_max_ deviations (between case and control days) within a limited period of one month.

The observed lag structure with main apparent effects on cause-specific admissions occurring within 5 days in our study is compatible with the patterns observed elsewhere [8]–[12]. However, few of these studies investigated confounding by air pollutants [8]–[10]. There is evidence that confounding by PM_10_ and NO_2max_ is present in the association between Tapp_max_ and RD, CVD or CBD hospital admissions, particularly in the warm period (Text S1). This finding has implications for the analyses of future studies.

Our estimate of 4% (95% CI: 2%, 6%) increase in total RD *emergency* admissions (>18 years) per 10 µg/m^3^ increase in the CA5 of PM_10_ concentration during the warm period, is slightly stronger than that of a previous study conducted in Copenhagen (six specific types of RD admission, ≥65 years, emergency or planned) and elsewhere [28]–[30].

Our estimate of association between Tapp_max_ and total CVD *emergency* admissions (>18 years) of 2% (0.3%; 4%) following 10 µg/m^3^ increase in the CA5 of PM_10_ during the warm period is generally higher than those observed from time-series studies in USA, Europe, and specifically in Stockholm (total CVD admissions; all ages, emergency or planned), and Copenhagen (ten specific types of CVD admissions, ≥65 years, emergency and planned) [28], [31], [32]. These studies also observed PM_10_ effects on CVD admissions within a week. We found no clear association between total CVD *emergency* admissions, and NO_2_, NO_2max_ or CO, suggesting weak influence of local traffic (Text S1).

The observed estimate associated with PM_10_ was stronger for RD than CVD admissions, suggesting a steeper dose-response relationship for the former. Other studies also observed higher estimates for PM_10_ effect on total RD morbidity than for total CVD [29]–[32].

Studies investigating modification of the effect of temperature on cause-specific hospital admissions are scarce. Sensitivity to temperature may be disease specific and understanding these differences may assist in identifying the vulnerable populations and planning of preventive measures. We found the strongest associations between Tapp_max_ in the warm period and RD or CVD admissions amongst women, the elderly, and the lowest SES group. Age has been reported as effect modifier for total RD admissions with the elderly more susceptible to temperature changes [8], [10].

The underlying mechanisms behind adverse effects of warm temperature for prolonged periods of time on CVD and RD outcomes may involve blood flow shifts to subcutaneous areas and away from the vital organs, in an effort to cool the body [2], [3]. Inadequate thermoregulation may occur when too much blood is diverted, putting increased stress on the heart and lungs. Increased blood viscosity due to dehydration and elevated cholesterol levels associated with higher temperatures, and a higher sweating threshold in the elderly may also trigger heat-related health effects in susceptible individuals [2], [3]. This is aggravated by any factor that hampers sweating, such as high ambient humidity, reduced air currents (no breeze, tight fitting clothes) or anticholinergic drugs [2], [3].

The mechanism by which cold ambient conditions can increase the risk of CVD remains unclear [4]. However, there are several factors which have been shown to have clear seasonal variations, including winter increases in plasma cholesterol, plasma fibrinogen, blood pressure, and red and white blood cell counts. In Copenhagen protective effects with respect to CVD of increasing temperature even in the warm season could be due to the relatively cold climate with average temperatures from 6°C in April to 16°C in July and yet more exposure as people tend to be more outdoors.

Advantages of our study include accurate meteorological, air pollution, and health outcome data. Some disease misclassification is possible, but it is unlikely to be related to temperature.

One limitation of the study is the assumption that the outdoor temperature and humidity measured in the inner city is the same across Greater Copenhagen. Another limitation is the assumption that outdoor temperature is a surrogate for personal exposure. This can potentially lead to bias in the estimated association, which may be more pronounced among the elderly and other frail groups who generally spend most of their time indoors and in fewer geographical areas.

Our results support the notion that moderate changes in ambient temperature are associated with impacts on human health even in a cool temperate climate. This association (assumed to be causal) is complex and depends on the specific health outcome (death or hospital admission), population characteristics (age, sex, SES), exposure conditions and the efficiency of the health care system, which all vary with time [33]. The results of this and many similar studies on temperature (and other key climate change factors) and health can thus not be extrapolated infinitely into the future without considering major uncertainties regarding changes in populations, the rate and intensity of projected climate change and adaptation, as stressed by the IPCC [6].

## Supporting Information

Figure S1 **Percentage change (95% CI) in cause-specific hospital admissions in Greater Copenhagen per IQR increase in Tapp_max_ during the warm and cold periods (1 January 2002−31 December 2006), adjusted for public holidays and influenza (not for any pollutants).**
(TIF)

Figure S2 **Percentage change (95% CI) in cause-specific hospital admissions in Greater Copenhagen per IQR increase in PM_10_, NO_2_, NO_2max_ and CO during the warm period (1 January 2002−31 December 2006), adjusted for Tapp_max_, public holidays and influenza.**
(TIF)

Figure S3 **Percentage change (95% CI) in cause-specific hospital admissions in Greater Copenhagen per IQR increase in PM_10_, NO_2_, NO_2max_ and CO during the cold period (1 January 2002−31 December 2006), adjusted for Tapp_max_, public holidays and influenza.**
(TIF)

Figure S4 **Percentage change (95% CI) in cause-specific hospital admissions in Greater Copenhagen per IQR increase in Tapp_max_ during the warm period (1 January 2002 − 31 December 2006), adjusted for public holidays, influenza and PM_10_, NO_2_, NO_2max_ or CO.**
(TIF)

Figure S5 **Percentage change (95% CI) in cause-specific hospital admissions in Greater Copenhagen per IQR increase in Tapp_max_ during the cold period (1 January 2002−31 December 2006), adjusted for public holidays, influenza and PM_10_, NO_2_, NO_2max_ or CO.**
(TIF)

Figure S6 **Percentage change (95% CI) in cause-specific hospital admissions in Greater Copenhagen per IQR increase in Tapp_max_ during the cold period (1 January 2002−31 December 2006), adjusted for public holidays and influenza (CVD and CBD), and PM_10_ (RD).**
(TIF)

Figure S7 **Total and average number of cause-specific hospital admissions per Tapp_max_ (lag0) in Greater Copenhagen during 1 January 2002−31 December 2006.**
(TIF)

Figure S8 **Percentage change (95% CI) in cause-specific hospital admissions in Greater Copenhagen per IQR increase in temperature during the warm and cold periods (1 January 2002−31 December 2006), adjusted for public holidays, influenza and relative humidity (not for any pollutants).**
(TIF)

Figure S9 **Percentage change (95% CI) in cause-specific hospital admissions in Greater Copenhagen per IQR increase in temperature during the warm and cold periods (1 January 2002−31 December 2006), adjusted for public holidays and influenza (not for relative humidity or any pollutants).**
(TIF)

Table S1 **Association between Tapp_max_ and total respiratory hospital admissions expressed as percentage increase in risk (%) and 95% confidence intervals per inter-quartile increase in 5-day cumulative average of Tapp_max_ (in °C) and 5-day cumulative average of PM_10_ (in µg.m^−3^) and NO_2_ (in ppb) during the warm and cold period of 1 January 2002−31 December 2006 in Greater Copenhagen.**
(DOC)

Table S2 **Association between Tapp_max_ and total cardiovascular hospital admissions expressed as percentage increase in risk (%) and 95% confidence intervals per inter-quartile increase in 5-day cumulative average of Tapp_max_ (in °C) and 5-day cumulative average of PM_10_ (in µg.m^−3^), NO_2_ (in ppb), NO_2max_ (in ppb) and CO (in ppm) during the warm period of 1 January 2002−31 December 2006 in Greater Copenhagen.**
(DOC)

Table S3 **Association between Tapp_max_ and total cerebrovascular hospital admissions expressed as percentage increase in risk (%) and 95% confidence intervals per inter-quartile increase in 5-day cumulative average of Tapp_max_ (in °C) and 5-day cumulative average of NO_2max_ (in ppb) during the warm period of 1 January 2002−31 December 2006 in Greater Copenhagen.**
(DOC)

Table S4 **Association between temperature and hospital admissions, by cause, expressed as percentage increase in risk (%) and 95% confidence intervals per inter-quartile increase in 5-day cumulative average of temperature (in °C) during the warm period of 1 January 2002−31 December 2006 in Greater Copenhagen.**
(DOC)

Table S5 **Association between temperature and hospital admissions, by cause, expressed as percentage increase in risk (%) and 95% confidence intervals per inter-quartile increase in 5-day cumulative average of temperature (in °C) during the cold period of 1 January 2002−31 December 2006 in Greater Copenhagen.**
(DOC)

Table S6 **Association between temperature and hospital admissions, by cause, expressed as percentage increase in risk (%) and 95% confidence intervals per inter-quartile increase in 5-day cumulative average of temperature (in °C) during the warm period of 1 January 2002−31 December 2006 in Greater Copenhagen.**
(DOC)

Table S7 **Association between temperature and hospital admissions, by cause, expressed as percentage increase in risk (%) and 95% confidence intervals per inter-quartile increase in 5-day cumulative average of temperature (in °C) during the cold period of 1 January 2002−31 December 2006 in Greater Copenhagen.**
(DOC)

Table S8 **Spearman correlation coefficient between temperature, relative humidity and pollutants in Greater Copenhagen during 1 January 2002−31 December 2006.**
(DOC)

Text S1 **Lag selection of Tapp_max_ and air pollutants.**
(DOC)

Text S2 **Longer lags in the cold period.**
(DOC)

Text S3 **Tapp_max_ threshold.**
(DOC)

Text S4 **Sensitivity analyses: alternative temperature definition.**
(DOC)
