# Supplementary figures and images for: Apparent Temperature and Cause-Specific Emergency Hospital Admissions in Greater Copenhagen, Denmark
Source: PLoS One. 2011 Jul 29;6(7):e22904. doi: 10.1371/journal.pone.0022904 (PMC3146500; doi:10.1371/journal.pone.0022904)

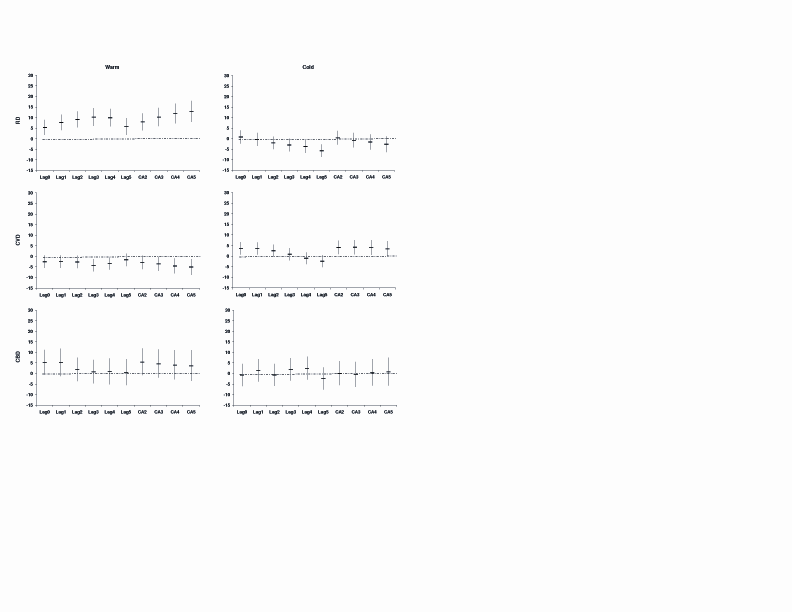

Supplement: Figure S1 — Percentage change (95% CI) in cause-specific hospital admissions in Greater Copenhagen per IQR increase in Tappmax during the warm and cold periods (1 January 2002−31 December 2006), adjusted for public holidays and influenza (not for any pollutants). (TIF) [file pone.0022904.s001.tif]

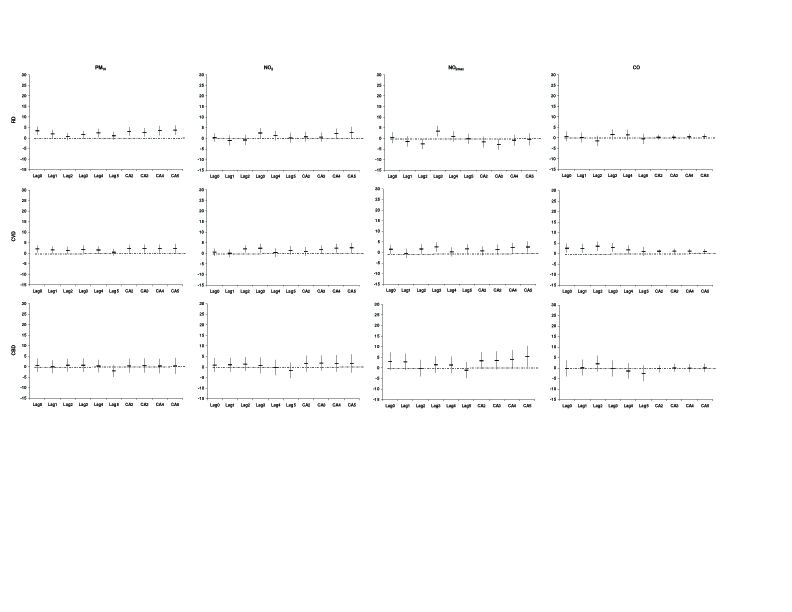

Supplement: Figure S2 — Percentage change (95% CI) in cause-specific hospital admissions in Greater Copenhagen per IQR increase in PM10, NO2, NO2max and CO during the warm period (1 January 2002−31 December 2006), adjusted for Tappmax, public holidays and influenza. (TIF) [file pone.0022904.s002.tif]

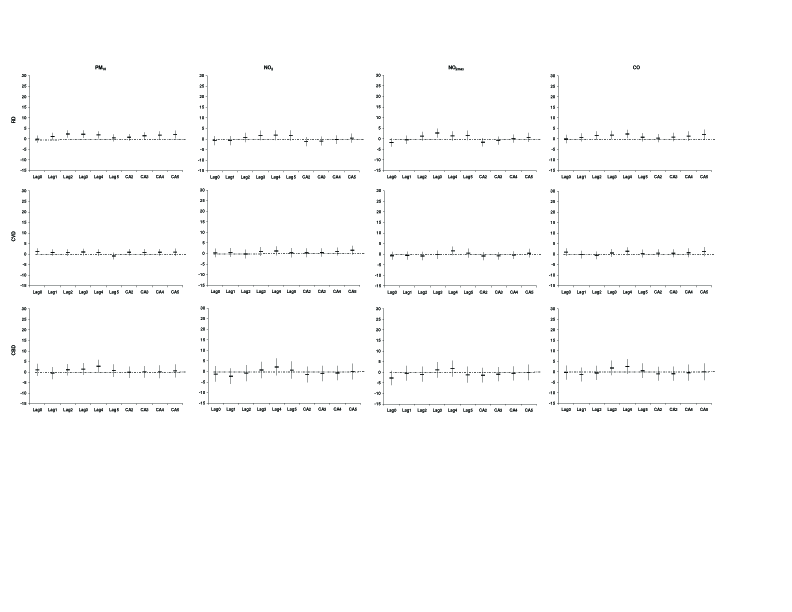

Supplement: Figure S3 — Percentage change (95% CI) in cause-specific hospital admissions in Greater Copenhagen per IQR increase in PM10, NO2, NO2max and CO during the cold period (1 January 2002−31 December 2006), adjusted for Tappmax, public holidays and influenza. (TIF) [file pone.0022904.s003.tif]

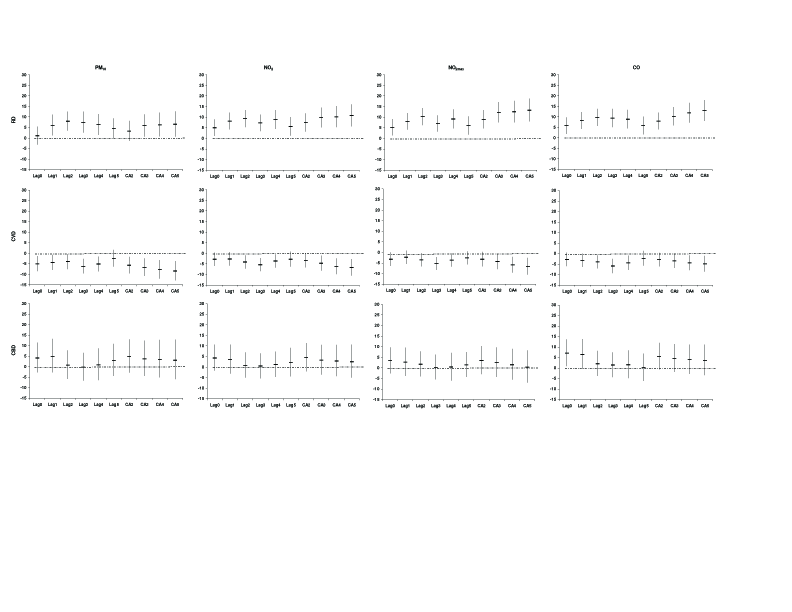

Supplement: Figure S4 — Percentage change (95% CI) in cause-specific hospital admissions in Greater Copenhagen per IQR increase in Tappmax during the warm period (1 January 2002 − 31 December 2006), adjusted for public holidays, influenza and PM10, NO2, NO2max or CO. (TIF) [file pone.0022904.s004.tif]

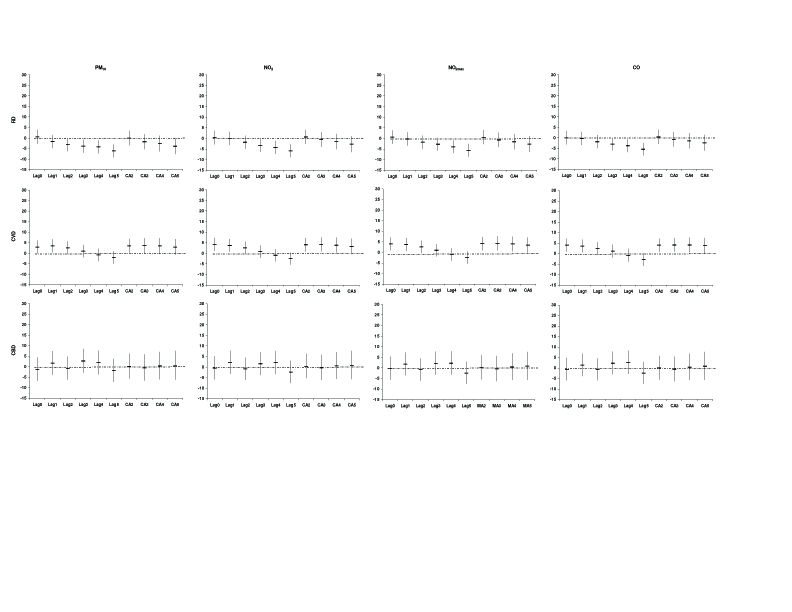

Supplement: Figure S5 — Percentage change (95% CI) in cause-specific hospital admissions in Greater Copenhagen per IQR increase in Tappmax during the cold period (1 January 2002−31 December 2006), adjusted for public holidays, influenza and PM10, NO2, NO2max or CO. (TIF) [file pone.0022904.s005.tif]

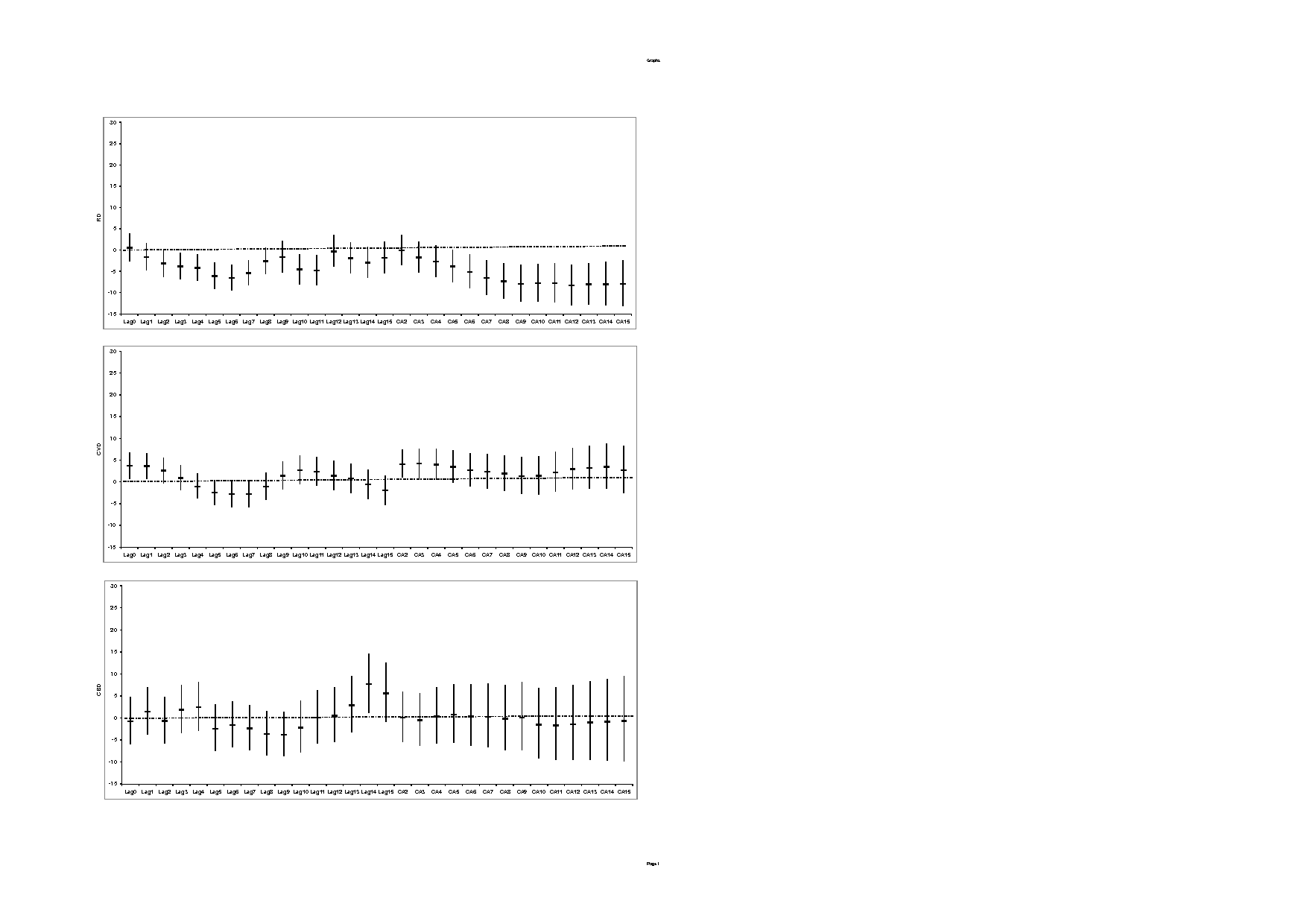

Supplement: Figure S6 — Percentage change (95% CI) in cause-specific hospital admissions in Greater Copenhagen per IQR increase in Tappmax during the cold period (1 January 2002−31 December 2006), adjusted for public holidays and influenza (CVD and CBD), and PM10 (RD). (TIF) [file pone.0022904.s006.tif]

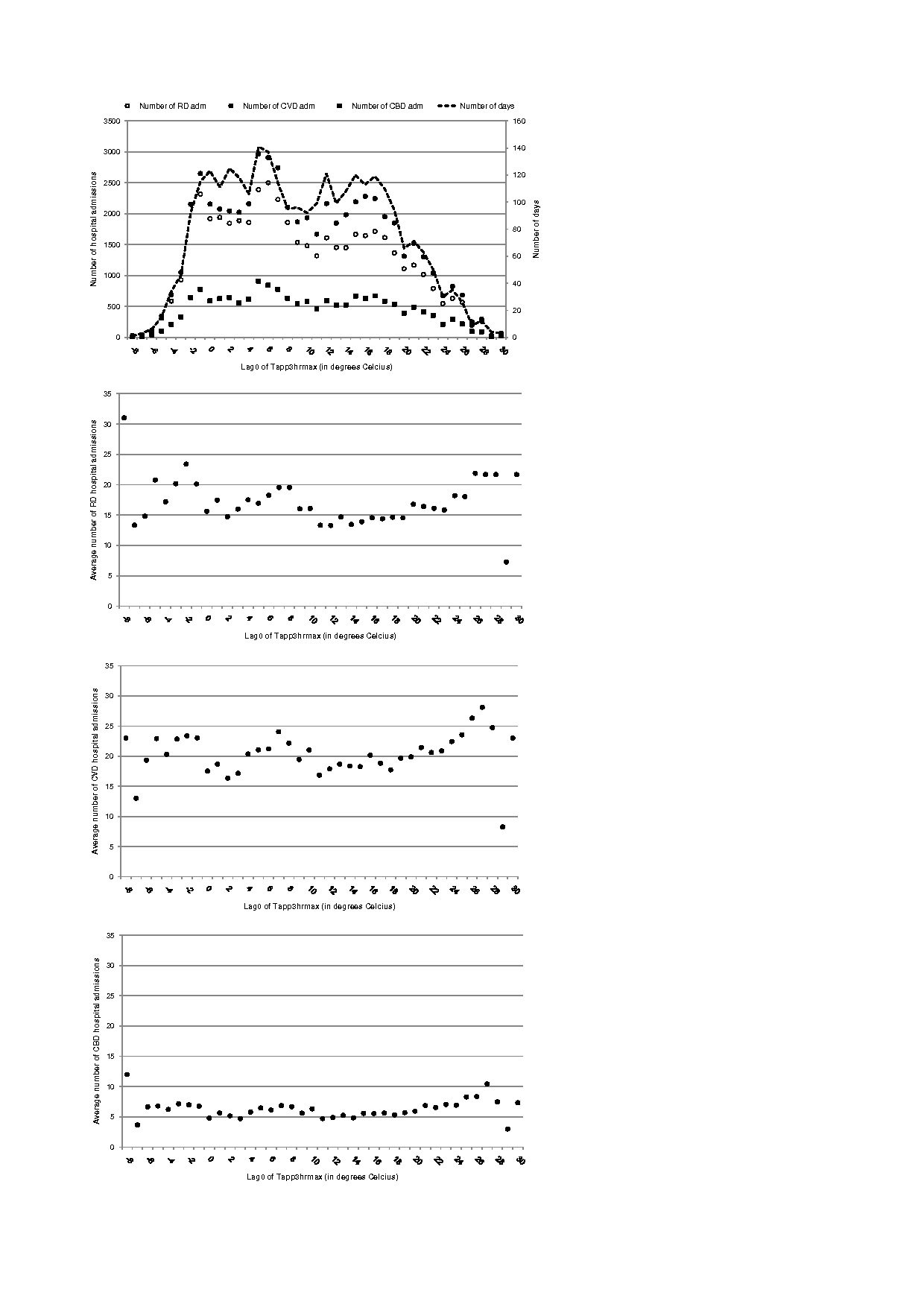

Supplement: Figure S7 — Total and average number of cause-specific hospital admissions per Tappmax (lag0) in Greater Copenhagen during 1 January 2002−31 December 2006. (TIF) [file pone.0022904.s007.tif]

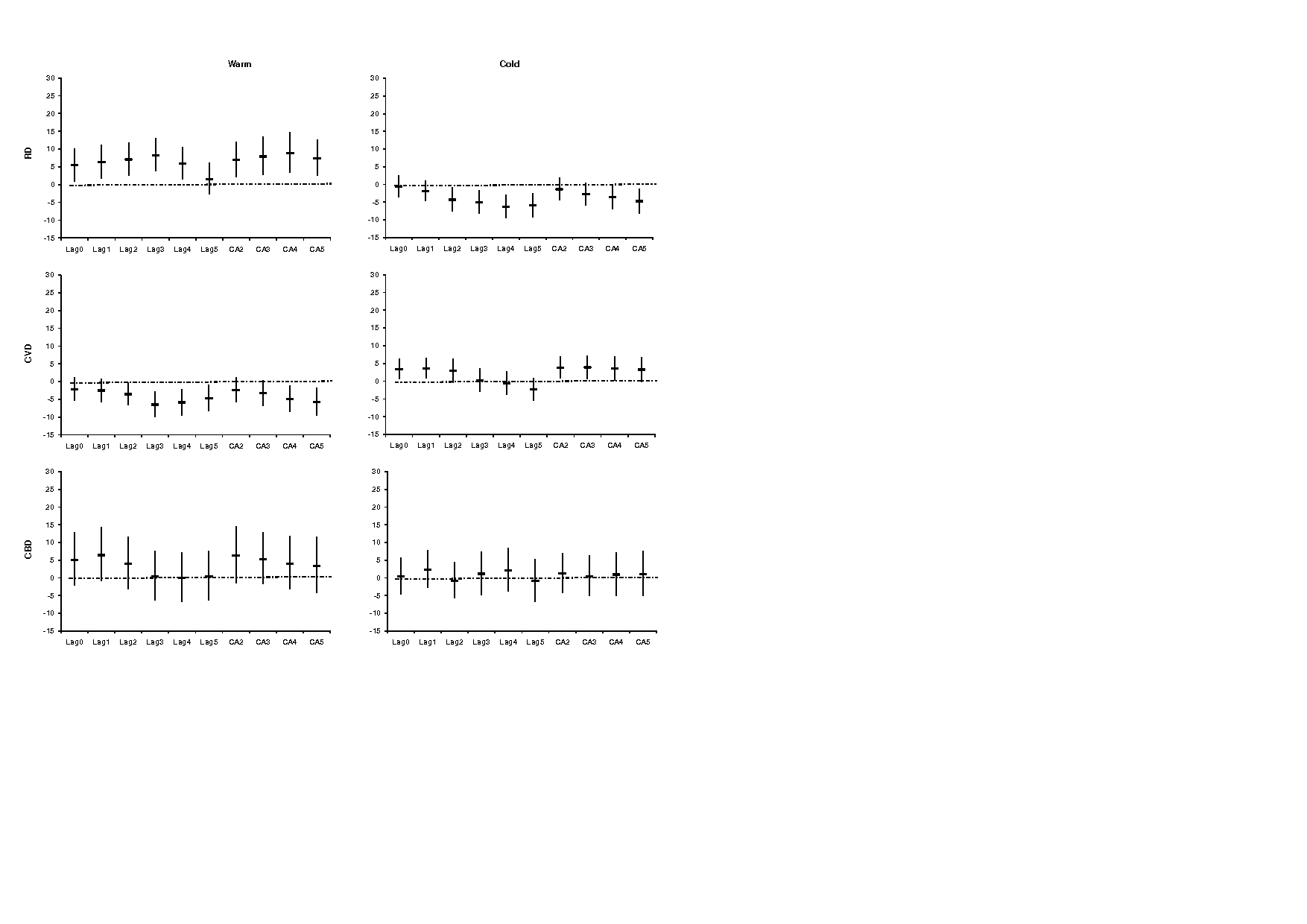

Supplement: Figure S8 — Percentage change (95% CI) in cause-specific hospital admissions in Greater Copenhagen per IQR increase in temperature during the warm and cold periods (1 January 2002−31 December 2006), adjusted for public holidays, influenza and relative humidity (not for any pollutants). (TIF) [file pone.0022904.s008.tif]

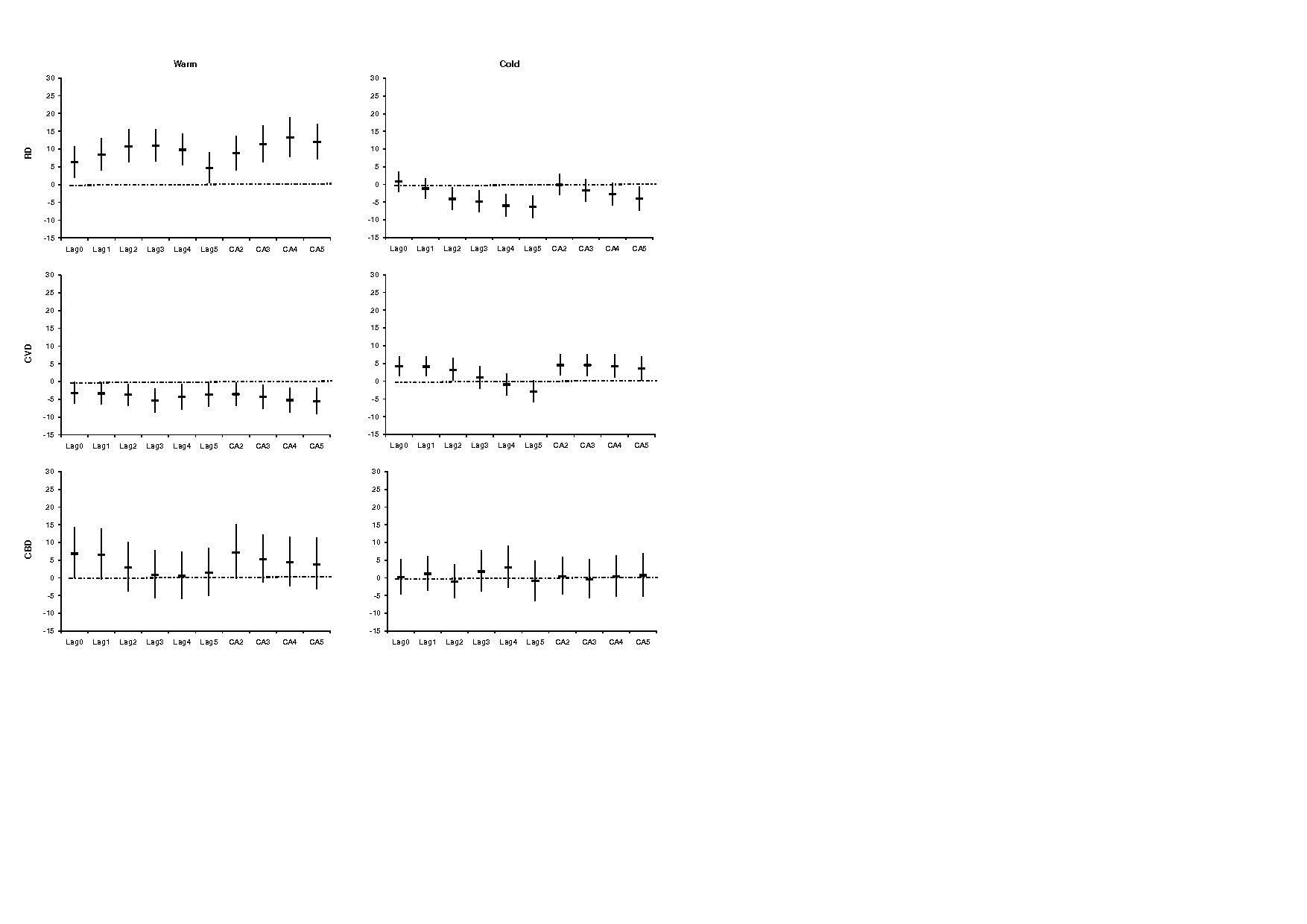

Supplement: Figure S9 — Percentage change (95% CI) in cause-specific hospital admissions in Greater Copenhagen per IQR increase in temperature during the warm and cold periods (1 January 2002−31 December 2006), adjusted for public holidays and influenza (not for relative humidity or any pollutants). (TIF) [file pone.0022904.s009.tif]
