# Supplementary material for: Apparent Temperature and Cause-Specific Emergency Hospital Admissions in Greater Copenhagen, Denmark
Source: PLoS One. 2011 Jul 29;6(7):e22904. doi: 10.1371/journal.pone.0022904 (PMC3146500; doi:10.1371/journal.pone.0022904)
Supplement: Table S1 — Association between Tappmax and total respiratory hospital admissions expressed as percentage increase in risk (%) and 95% confidence intervals per inter-quartile increase in 5-day cumulative average of Tappmax (in °C) and 5-day cumulative average of PM10 (in µg.m−3) and NO2 (in ppb) during the warm and cold period of 1 January 2002−31 December 2006 in Greater Copenhagen. (DOC) [file pone.0022904.s010.doc]

**Table S1. Association between Tapp_max_ and total respiratory hospital admissions expressed as percentage increase in risk (%) and 95% confidence intervals per inter-quartile increase in 5-day cumulative average of Tapp_max_ (in °C) and 5-day cumulative average of PM_10_ (in µg.m^-3^) and NO_2_ (in ppb) during the warm and cold period of 1 January 2002−31 December 2006 in Greater Copenhagen.**

|  | **Warm period** | | | | | **Cold period** | | | | |
| --- | --- | --- | --- | --- | --- | --- | --- | --- | --- | --- |
|  | **IQR** | **n^a^** | **%** | **95% CI** | | **IQR** | **n** | **%** | **95% CI** | |
| Model 1 |  |  |  |  |  |  |  |  |  |  |
| Tapp_max_ | 8 | 20836 | **12.9** | **8.1** | **17.9** | 7 | 24114 | -2.7 | -6.3 | 1.1 |
| Model 2 |  |  |  |  |  |  |  |  |  |  |
| Tapp_max_ | 8 | 20350 | **6.5** | **0.7** | **12.6** | 7 | 22593 | -3.9 | -7.6 | 0.0 |
| PM_10_ | 10 | 20350 | **3.8** | **1.5** | **6.1** | 12 | 22593 | **2.0** | **0.2** | **3.9** |
| Model 3 |  |  |  |  |  |  |  |  |  |  |
| Tapp_max_ | 8 | 19491 | **6.5** | **0.6** | **12.6** | 7 | 22174 | -3.9 | -7.7 | 0.0 |
| PM_10_ | 10 | 19491 | **3.3** | **0.8** | **5.9** | 12 | 22174 | **2.5** | **0.4** | **4.6** |
| NO_2_ | 4 | 19491 | 1.0 | -1.8 | 4.0 | 5 | 22174 | -1.0 | -3.5 | 1.5 |

Warm period: April–September, Cold period: October–March

Models adjusted for public holidays and influenza rates.

^a^Number of admissions
