# Supplementary material for: Apparent Temperature and Cause-Specific Emergency Hospital Admissions in Greater Copenhagen, Denmark
Source: PLoS One. 2011 Jul 29;6(7):e22904. doi: 10.1371/journal.pone.0022904 (PMC3146500; doi:10.1371/journal.pone.0022904)
Supplement: Table S2 — Association between Tappmax and total cardiovascular hospital admissions expressed as percentage increase in risk (%) and 95% confidence intervals per inter-quartile increase in 5-day cumulative average of Tappmax (in °C) and 5-day cumulative average of PM10 (in µg.m−3), NO2 (in ppb), NO2max (in ppb) and CO (in ppm) during the warm period of 1 January 2002−31 December 2006 in Greater Copenhagen. (DOC) [file pone.0022904.s011.doc]

**Table S2. Association between Tapp_max_ and total cardiovascular hospital admissions expressed as percentage increase in risk (%) and 95% confidence intervals per inter-quartile increase in 5-day cumulative average of Tapp_max_ (in °C) and 5-day cumulative average of PM_10_ (in µg.m^-3^), NO_2_ (in ppb), NO_2max_ (in ppb) and CO (in ppm) during the warm period of 1 January 2002−31 December 2006 in Greater Copenhagen.**

|  | **IQR** | **n^a^** | **%** | **95% CI** | |
| --- | --- | --- | --- | --- | --- |
| Model 1 |  |  |  |  |  |
| Tapp_max_ | 8 | 26530 | **-5.1** | **-8.7** | **-1.3** |
| Model 2 |  |  |  |  |  |
| Tapp_max_ | 8 | 25872 | **-8.4** | **-12.9** | **-3.7** |
| PM_10_ | 10 | 25872 | **2.3** | **0.3** | **4.4** |
| Model 3 |  |  |  |  |  |
| Tapp_max_ | 7 | 25601 | **-5.9** | **-9.3** | **-2.4** |
| NO_2_ | 4 | 25601 | **2.6** | **0.3** | **5.0** |
| Model 4 |  |  |  |  |  |
| Tapp_max_ | 7 | 25732 | **-5.8** | **-9.2** | **-2.3** |
| NO_2max_ | 9 | 25732 | **2.6** | **0.0** | **5.2** |
| Model 5 |  |  |  |  |  |
| Tapp_max_ | 8 | 24436 | **-4.9** | **-8.5** | **-1.1** |
| CO | 0.065 | 24436 | **1.0** | **0.0** | **2.1** |
| Model 6 |  |  |  |  |  |
| Tapp_max_ | 8 | 24943 | **-8.5** | **-13.0** | **-3.8** |
| PM_10_ | 9 | 24943 | 1.5 | -0.6 | 3.6 |
| NO_2_ | 4 | 24943 | 1.8 | -0.8 | 4.5 |
| Model 7 |  |  |  |  |  |
| Tapp_max_ | 8 | 25074 | **-9.0** | **-13.5** | **-4.2** |
| PM_10_ | 10 | 25074 | 1.9 | -0.2 | 4.1 |
| NO_2max_ | 9 | 25074 | 1.9 | -0.7 | 4.7 |
| Model 8 |  |  |  |  |  |
| Tapp_max_ | 8 | 23778 | **-7.8** | **-12.4** | **-3.0** |
| PM_10_ | 10 | 23778 | 2.0 | -0.1 | 4.1 |
| CO | 0.066 | 23778 | 0.8 | -0.2 | 1.8 |
| Model 9 |  |  |  |  |  |
| Tapp_max_ | 7 | 23874 | **-5.6** | **-9.0** | **-2.0** |
| NO_2_ | 4 | 23874 | 2.3 | -0.1 | 4.7 |
| CO | 0.066 | 23874 | 0.8 | -0.2 | 1.8 |
| Model 10 |  |  |  |  |  |
| Tapp_max_ | 8 | 23932 | **-6.3** | **-10.2** | **-2.2** |
| NO_2max_ | 9 | 23932 | 2.3 | -0.3 | 5.0 |
| CO | 0.066 | 23932 | 0.9 | -0.1 | 1.9 |

Warm period: April–September

Models adjusted for public holidays and influenza rates.

^a^Number of admissions
