# Supplementary material for: Apparent Temperature and Cause-Specific Emergency Hospital Admissions in Greater Copenhagen, Denmark
Source: PLoS One. 2011 Jul 29;6(7):e22904. doi: 10.1371/journal.pone.0022904 (PMC3146500; doi:10.1371/journal.pone.0022904)
Supplement: Table S3 — Association between Tappmax and total cerebrovascular hospital admissions expressed as percentage increase in risk (%) and 95% confidence intervals per inter-quartile increase in 5-day cumulative average of Tappmax (in °C) and 5-day cumulative average of NO2max (in ppb) during the warm period of 1 January 2002−31 December 2006 in Greater Copenhagen. (DOC) [file pone.0022904.s012.doc]

**Table S3. Association between Tapp_max_ and total cerebrovascular hospital admissions expressed as percentage increase in risk (%) and 95% confidence intervals per inter-quartile increase in 5-day cumulative average of Tapp_max_ (in °C) and 5-day cumulative average of NO_2max_ (in ppb) during the warm period of 1 January 2002−31 December 2006 in Greater Copenhagen.**

|  | **IQR** | **n^a^** | **%** | **95% CI** | |
| --- | --- | --- | --- | --- | --- |
| Model 1 |  |  |  |  |  |
| Tapp_max_ | 8 | 7995 | 3.6 | -3.5 | 11.1 |
| Model 2 |  |  |  |  |  |
| Tapp_max_ | 8 | 7762 | 0.3 | -7.0 | 8.2 |
| NO_2max_ | 9 | 7762 | **5.4** | **0.6** | **10.3** |

Warm period: April–September

Models adjusted for public holidays and influenza rates.

^a^Number of admissions
