# Supplementary material for: Apparent Temperature and Cause-Specific Emergency Hospital Admissions in Greater Copenhagen, Denmark
Source: PLoS One. 2011 Jul 29;6(7):e22904. doi: 10.1371/journal.pone.0022904 (PMC3146500; doi:10.1371/journal.pone.0022904)
Supplement: Table S5 — Association between temperature and hospital admissions, by cause, expressed as percentage increase in risk (%) and 95% confidence intervals per inter-quartile increase in 5-day cumulative average of temperature (in °C) during the cold period of 1 January 2002−31 December 2006 in Greater Copenhagen. (DOC) [file pone.0022904.s014.doc]

**Table S5. Association between temperature and hospital admissions, by cause, expressed as percentage increase in risk (%) and 95% confidence intervals per inter-quartile increase in 5-day cumulative average of temperature (in °C) during the cold period of 1 January 2002−31 December 2006 in Greater Copenhagen.**

|  | **Respiratory disease^a.b^** | | | | | **Cardiovascular disease^a.b^** | | | | | **Cerebrovascular disease^a^** | | | | |
| --- | --- | --- | --- | --- | --- | --- | --- | --- | --- | --- | --- | --- | --- | --- | --- |
|  | **n^c^** | **IQR** | **%** | **95% CI** | | **n** | **IQR** | **%** | **95% CI** | | **n** | **IQR** | **%** | **95% CI** | |
| **All** | 22593 | 6 | **-7.0** | **-10.6** | **-3.3** | 27911 | 6 | 3.3 | -0.2 | 6.8 | 8064 | 6 | 1.1 | -5.0 | 7.7 |
| **Age categories** |  |  |  |  |  |  |  |  |  |  |  |  |  |  |  |
| 19-65 years | 5551 | 6 | -0.8 | -8.2 | 7.4 | 8988 | 7 | 2.3 | -4.6 | 9.7 | 2056 | 6 | 9.0 | -3.7 | 23.3 |
| 66-80 years | 9789 | 6 | -4.3 | -9.8 | 1.6 | 10130 | 6 | 4.3 | -1.3 | 10.2 | 3121 | 6 | 1.2 | -8.5 | 11.9 |
| > 80 years | 7253 | 6 | **-15.1** | **-20.9** | **-9.0** | 8793 | 6 | 3.2 | -2.8 | 9.6 | 2887 | 6 | -4.4 | -14.0 | 6.2 |
| **Sex** |  |  |  |  |  |  |  |  |  |  |  |  |  |  |  |
| Women | 12982 | 6 | **-8.3** | **-12.9** | **-3.4** | 12785 | 6 | -0.5 | -5.3 | 4.5 | 4199 | 6 | 1.4 | -7.1 | 10.7 |
| Men | 9611 | 6 | -5.3 | -10.9 | 0.6 | 15126 | 6 | **6.8** | **2.0** | **11.9** | 3865 | 6 | 1.0 | -7.7 | 10.4 |
| **Socio-economic status** |  |  |  |  |  |  |  |  |  |  |  |  |  |  |  |
| Lowest | 7605 | 6 | -6.3 | -12.4 | 0.2 | 8471 | 6 | 3.3 | -2.9 | 9.8 | 1855 | 6 | **14.0** | **0.2** | **29.7** |
| Second lowest | 5563 | 6 | **-7.8** | **-14.7** | **-0.3** | 7414 | 6 | 0.5 | -5.9 | 7.2 | 2291 | 6 | 0.3 | -10.8 | 12.9 |
| Second highest | 5015 | 6 | -5.2 | -12.8 | 3.1 | 6550 | 6 | **9.0** | **1.7** | **16.8** | 2043 | 6 | 4.2 | -8.3 | 18.5 |
| Highest | 2324 | 6 | -4.1 | -15.1 | 8.2 | 3329 | 6 | -5.9 | -14.6 | 3.8 | 1293 | 6 | -5.9 | -19.2 | 9.5 |

Cold period: October−March

^a^Adjusted for 5-day cumulative average of relative humidity, public holidays and influenza rates

^b^Adjusted for 5-day cumulative average of PM_10_ ((lag0 + lag1 + lag2 + lag3 + lag4)/5)

^c^Number of admissions
