# Supplementary material for: Apparent Temperature and Cause-Specific Emergency Hospital Admissions in Greater Copenhagen, Denmark
Source: PLoS One. 2011 Jul 29;6(7):e22904. doi: 10.1371/journal.pone.0022904 (PMC3146500; doi:10.1371/journal.pone.0022904)
Supplement: Table S6 — Association between temperature and hospital admissions, by cause, expressed as percentage increase in risk (%) and 95% confidence intervals per inter-quartile increase in 5-day cumulative average of temperature (in °C) during the warm period of 1 January 2002−31 December 2006 in Greater Copenhagen. (DOC) [file pone.0022904.s015.doc]

**Table S6. Association between temperature and hospital admissions, by cause, expressed as percentage increase in risk (%) and 95% confidence intervals per inter-quartile increase in 5-day cumulative average of temperature (in °C) during the warm period of 1 January 2002−31 December 2006 in Greater Copenhagen.**

|  | **Respiratory disease^a.b^** | | | | | **Cardiovascular disease^a.b^** | | | | | **Cerebrovascular disease^a.c^** | | | | |
| --- | --- | --- | --- | --- | --- | --- | --- | --- | --- | --- | --- | --- | --- | --- | --- |
|  | **n^d^** | **IQR** | **%** | **95% CI** | | **n** | **IQR** | **%** | **95% CI** | | **n** | **IQR** | **%** | **95% CI** | |
| **All** | 20350 | 7 | **6.9** | **0.4** | **13.9** | 25872 | 6 | **-8.5** | **-12.8** | **-3.9** | 7762 | 6 | 1.4 | -6.1 | 9.4 |
| **Age categories** |  |  |  |  |  |  |  |  |  |  |  |  |  |  |  |
| 19-65 years | 5036 | 6 | 2.7 | -8.0 | 14.6 | 8511 | 6 | **-8.7** | **-16.1** | **-0.5** | 2032 | 6 | -0.3 | -14.3 | 16.1 |
| 66-80 years | 8925 | 7 | **11.5** | **1.4** | **22.7** | 9497 | 6 | -1.8 | -9.4 | 6.4 | 2888 | 6 | 0.8 | -11.0 | 14.2 |
| > 80 years | 6389 | 7 | 4.0 | -7.1 | 16.3 | 7864 | 6 | **-15.6** | **-22.8** | **-7.8** | 2842 | 6 | 2.9 | -9.2 | 16.6 |
| **Sex** |  |  |  |  |  |  |  |  |  |  |  |  |  |  |  |
| Women | 11472 | 7 | **11.0** | **2.1** | **20.8** | 11740 | 6 | **-12.3** | **-18.5** | **-5.7** | 4094 | 6 | 2.0 | -8.0 | 13.1 |
| Men | 8878 | 7 | 1.9 | -7.4 | 12.1 | 14132 | 6 | -5.1 | -11.2 | 1.4 | 3668 | 6 | 0.6 | -10.1 | 12.6 |
| **Socio-economic status** |  |  |  |  |  |  |  |  |  |  |  |  |  |  |  |
| Lowest | 6819 | 7 | **12.7** | **1.0** | **25.7** | 7975 | 6 | **-17.0** | **-24.0** | **-9.4** | 1811 | 6 | 7.4 | -8.7 | 26.5 |
| Second lowest | 5287 | 6 | -1.8 | -11.6 | 9.2 | 6807 | 6 | -3.7 | -12.5 | 5.9 | 2154 | 7 | 12.0 | -5.5 | 32.8 |
| Second highest | 4572 | 6 | 9.2 | -2.7 | 22.5 | 6053 | 6 | -4.1 | -13.3 | 6.1 | 2053 | 6 | -6.0 | -18.8 | 8.8 |
| Highest | 1909 | 7 | 3.8 | -15.3 | 27.1 | 3065 | 6 | 2.7 | -11.0 | 18.4 | 1273 | 6 | -0.1 | -16.8 | 19.9 |

Warm period: April−September

^a^Adjusted for public holidays and influenza rates, but not for relative humidity.

^b^Adjusted for 5-day cumulative average of PM_10_ ((lag0 + lag1 + lag2 + lag3 + lag4)/5)

^c^Adjusted for 5-day cumulative average of NO_2max_

^d^Number of admissions
