# Supplementary material for: Apparent Temperature and Cause-Specific Emergency Hospital Admissions in Greater Copenhagen, Denmark
Source: PLoS One. 2011 Jul 29;6(7):e22904. doi: 10.1371/journal.pone.0022904 (PMC3146500; doi:10.1371/journal.pone.0022904)
Supplement: Table S7 — Association between temperature and hospital admissions, by cause, expressed as percentage increase in risk (%) and 95% confidence intervals per inter-quartile increase in 5-day cumulative average of temperature (in °C) during the cold period of 1 January 2002−31 December 2006 in Greater Copenhagen. (DOC) [file pone.0022904.s016.doc]

**Table S7. Association between temperature and hospital admissions, by cause, expressed as percentage increase in risk (%) and 95% confidence intervals per inter-quartile increase in 5-day cumulative average of temperature (in °C) during the cold period of 1 January 2002−31 December 2006 in Greater Copenhagen.**

|  | **Respiratory disease^a.b^** | | | | | **Cardiovascular disease^a.b^** | | | | | **Cerebrovascular disease^a^** | | | | |
| --- | --- | --- | --- | --- | --- | --- | --- | --- | --- | --- | --- | --- | --- | --- | --- |
|  | **n^c^** | **IQR** | **%** | **95% CI** | | **n** | **IQR** | **%** | **95% CI** | | **n** | **IQR** | **%** | **95% CI** | |
| **All** | 22593 | 6 | **-6.1** | **-9.6** | **-2.5** | 27911 | 6 | **3.6** | **0.3** | **7.0** | 8064 | 6 | 0.7 | -5.2 | 7.0 |
| **Age categories** |  |  |  |  |  |  |  |  |  |  |  |  |  |  |  |
| 19-65 years | 5551 | 6 | -1.4 | -8.5 | 6.3 | 8988 | 7 | 4.8 | -1.9 | 12.1 | 2056 | 6 | 9.4 | -2.8 | 23.2 |
| 66-80 years | 9789 | 6 | -4.6 | -9.9 | 1.0 | 10130 | 6 | 3.5 | -1.9 | 9.2 | 3121 | 6 | -0.3 | -9.5 | 9.8 |
| > 80 years | 7253 | 6 | **-11.8** | **-17.5** | **-5.6** | 8793 | 6 | 3.1 | -2.6 | 9.3 | 2887 | 6 | -4.1 | -13.3 | 6.2 |
| **Sex** |  |  |  |  |  |  |  |  |  |  |  |  |  |  |  |
| Women | 12982 | 6 | **-6.7** | **-11.2** | **-1.9** | 12785 | 6 | 0.8 | -3.8 | 5.7 | 4199 | 6 | 0.6 | -7.6 | 9.4 |
| Men | 9611 | 6 | -5.5 | -10.9 | 0.1 | 15126 | 6 | **6.1** | **1.5** | **10.9** | 3865 | 6 | 1.0 | -7.4 | 10.0 |
| **Socio-economic status** |  |  |  |  |  |  |  |  |  |  |  |  |  |  |  |
| Lowest | 7605 | 6 | -5.3 | -11.2 | 1.0 | 8471 | 6 | 4.2 | -1.8 | 10.4 | 1855 | 6 | 11.8 | -1.2 | 26.6 |
| Second lowest | 5563 | 6 | -7.3 | -14.0 | 0.0 | 7414 | 6 | 0.1 | -5.9 | 6.6 | 2291 | 6 | -1.6 | -12.2 | 10.2 |
| Second highest | 5015 | 6 | -4.8 | -12.1 | 3.2 | 6550 | 6 | **10.1** | **3.0** | **17.6** | 2043 | 6 | 4.7 | -7.3 | 18.4 |
| Highest | 2324 | 6 | -0.9 | -11.9 | 11.5 | 3329 | 6 | -6.3 | -14.7 | 3.0 | 1293 | 6 | -5.4 | -18.4 | 9.6 |

Cold period: October−March

^a^Adjusted for public holidays and influenza rates, but not for relative humidity.

^b^Adjusted for 5-day cumulative average of PM_10_ ((lag0 + lag1 + lag2 + lag3 + lag4)/5)

^c^Number of admissions
