# Supplementary material for: Apparent Temperature and Cause-Specific Emergency Hospital Admissions in Greater Copenhagen, Denmark
Source: PLoS One. 2011 Jul 29;6(7):e22904. doi: 10.1371/journal.pone.0022904 (PMC3146500; doi:10.1371/journal.pone.0022904)
Supplement: Table S8 — Spearman correlation coefficient between temperature, relative humidity and pollutants in Greater Copenhagen during 1 January 2002−31 December 2006. (DOC) [file pone.0022904.s017.doc]

**Table S8. Spearman correlation coefficient between temperature, relative humidity and pollutants in Greater Copenhagen during 1 January 2002−31 December 2006.**

| **Cold period** | **RH** | **PM_10_** | **CO** | **NO_2_** | **NO_2max_** |
| --- | --- | --- | --- | --- | --- |
| **Temperature** | 0.137^a^, 870^b^, <0.0001^c^ | 0.060, 854, 0.078 | -0.295, 855, <0.0001 | 0.011, 849, 0.751 | -0.039, 855, 0.253 |
| **RH** | - | 0.249, 854, <0.0001 | 0.185, 855, <0.0001 | 0.182, 849, <0.0001 | -0.023, 855, 0.506 |
| **PM_10_** | - | - | 0.549, 864, <0.0001 | 0.475, 859, <0.0001 | 0.294, 868, <0.0001 |
| **CO** | - | - | - | 0.707, 869, <0.0001 | 0.581, 870, <0.0001 |
| **Warm period** | **RH** | **PM_10_** | **CO** | **NO_2_** | **NO_2max_** |
| **Temperature** | -0.134, 889, <0.0001 | 0.278, 884, <0.0001 | -0.247, 823, <0.0001 | 0.042, 869, 0.218 | 0.072, 875, 0.033 |
| **RH** | - | 0.051, 884, 0.131 | 0.178, 823, <0.0001 | 0.084, 869, 0.013 | -0.002, 875, 0.950 |
| **PM_10_** | - | - | 0.462, 833, <0.0001 | 0.440, 881, <0.0001 | 0.358, 889, <0.0001 |
| **CO** | - | - | - | 0.571, 826, <0.0001 | 0.498, 830, <0.0001 |

Cold period: October–March, Warm period: April–September, RH: Relative humidity

^a^Spearman correlation coefficient

^b^Number of days with data

^c^p-value
