# Supplementary material for: Apparent Temperature and Cause-Specific Emergency Hospital Admissions in Greater Copenhagen, Denmark
Source: PLoS One. 2011 Jul 29;6(7):e22904. doi: 10.1371/journal.pone.0022904 (PMC3146500; doi:10.1371/journal.pone.0022904)
Supplement: Text S1 — Lag selection of Tappmax and air pollutants. (DOC) [file pone.0022904.s018.doc]

**Text S1**

**Lag selection of Tapp_max_ and air pollutants**

Figure S1 illustrates the % change in the cause-specific admissions per IQR increase in the different lags of Tapp_max_ during the warm and cold periods, respectively, after adjusting for public holidays and weekly influenza rates, but not for any of the pollutants. In general the strongest statistically significant association and model with the lowest Akaike Information Criterion (AIC) was observed between the 5-day cumulative average (CA5) of Tapp_max_ and the cause-specific admissions.

Figures S2 and S3 illustrate the % change in the cause-specific admissions per IQR increase in the different lags of PM_10_, NO_2_, NO_2max_ and CO during the warm and cold periods, respectively, after adjusting for Tapp_max_ (same lag as pollutant), public holidays and weekly influenza rates. In general the strongest significant association was observed between the CA5 of PM_10_ and NO_2_, and the RD and CVD admissions during the warm period (also models with lowest AIC). The CA5 of NO_2max_ was also associated with CVD admissions during the warm period. For CBD admissions in the warm period, the strongest significant association was observed between the CA5 of NO_2max_. None of the pollutants were associated with any of the cause-specific admissions during the cold period, expect for the CA5 of PM_10_ with RD admissions.

Figures S4 and S5 illustrate the % change in the cause-specific admissions per IQR increase in the different lags of Tapp_max_ during the warm and cold periods, respectively, after adjusting for the pollutants (same lag as Tapp_max_), public holidays and weekly influenza rates. In general the strongest statistically significant association was observed between the CA5 of Tapp_max_ and the cause-specific admissions during both periods (models with lowest AIC). In the warm period, the association between RD admissions and Tapp_max_ was weaker when controlled for PM_10_, but similar when controlled for NO_2_, NO_2max_ or CO (Figure S4). NO_2max_ and CO were insignificant in the 1-pollutant RD models in the warm period (Figure S2). Similar associations between RD admissions and Tapp_max_ were observed in both the 1-pollutant model (with PM_10_) and the 2-pollutant model (with PM_10_ and NO_2_) (Table S1). NO_2_ was insignificant in the 2-pollutant RD model and the RD models were therefore only adjusted for PM_10_ (Table 3).

In the warm period, the association between CVD admissions and Tapp_max_ was stronger when controlled for PM_10_, but similar when controlled for NO_2_, NO_2max_ or CO (Figure S4). Similar associations between CVD admissions and Tapp_max_ were observed in the 1-pollutant model (with PM_10_) and the 2-pollutant models (with PM_10_ and NO_2_, NO_2max_ or CO) in the warm period (Table S2). None of the pollutants were significant in the 2-pollutant CVD models during the warm period. The CVD models were therefore only adjusted for PM_10_ (Table 3).

Similar associations were observed between Tapp_max_ and CBD admissions after adjusting for the air pollutants in the warm period (Figure S4). NO_2max_ remained significant in the model and was adjusted for (Table S3 and Table 3).

In the cold period similar associations were observed between Tapp_max_ and the cause-specific admissions after adjusting for the air pollutants (Figure S5). The results in Table 4 were thus not adjusted for any pollutants, except for the RD models that were adjusted for PM_10_ (Table S1 and Table 4).
