# Supplementary material for: Apparent Temperature and Cause-Specific Emergency Hospital Admissions in Greater Copenhagen, Denmark
Source: PLoS One. 2011 Jul 29;6(7):e22904. doi: 10.1371/journal.pone.0022904 (PMC3146500; doi:10.1371/journal.pone.0022904)
Supplement: Text S2 — Longer lags in the cold period. (DOC) [file pone.0022904.s019.doc]

**Text S2**

**Longer lags in the cold period**

We investigated longer lags for Tapp_max_ in the cold period, after adjusting for public holidays, weekly influenza rates (CVD and CBD hospital admissions) and PM_10_ (RD hospital admissions) (Figure S6). From the literature it is known that extreme temperature effects follow shorter lags, whilst cold effects may have a lag of up to 15 days, at least for *mortality* [1]. We did not observe any stronger effects between Tapp_max_, and CVD or CBD hospital admissions after 5 days cumulative exposure. The effect of cold on RD hospital admissions appears to be slightly stronger after 5 days cumulative exposure (compare CA5 to CA9 of Tapp_max_), but the effect remains similar after 9 days cumulative exposure (compare CA9 to CA15 of Tapp_max_). We thus reported results for only up to 5 days cumulative exposure. We followed this approach when adjusting for PM_10_ in the RD hospital admission models: The same lag was used for PM_10_ and Tapp_max_ up to lag5, then lag5 for PM_10_ was used with lag6 to lag15 of Tapp_max_. The same cumulative average was used for PM_10_ and Tapp_max_ up to CA5, then CA5 for PM_10_ was used with CA6 to CA15 of Tapp_max_. We selected this approach as a previous study observed PM_10_ effects on hospital admissions within a week [2].

**References**

1. Analitis A, Katsouyanni K, Biggeri A, Baccini M, Forsberg B, et al (2008) Effects of cold weather on mortality: results from 15 European cities within the PHEWE project. Am Epidemiol 168(12):1397-1408.
2. Andersen ZJ, Wahlin P, Raaschou-Nielsen O, Scheike T, Loft S (2007) Ambient particle source apportionment and daily hospital admissions among children and elderly in Copenhagen. J Expo Sci Environ Epidemiol 17(7):625-636.
