# Supplementary material for: Apparent Temperature and Cause-Specific Emergency Hospital Admissions in Greater Copenhagen, Denmark
Source: PLoS One. 2011 Jul 29;6(7):e22904. doi: 10.1371/journal.pone.0022904 (PMC3146500; doi:10.1371/journal.pone.0022904)
Supplement: Text S3 — Tappmax threshold. (DOC) [file pone.0022904.s020.doc]

**Text S3**

**Tapp_max_ threshold**

Figure S7 indicates the average number of cause-specific hospital admissions per Tapp_max_ (lag0). We did not observe a Tapp_max_ threshold in Copenhagen for which a minimum number of cause-specific hospital admissions occurred. We therefore split a year into a warm and cold period. The warm and cold periods were defined as April–September and October–March, respectively, as for Stockholm and other European cities [1-3].

**References**

1. Analitis A, Katsouyanni K, Biggeri A, Baccini M, Forsberg B, et al (2008) Effects of cold weather on mortality: results from 15 European cities within the PHEWE project. Am . Epidemiol 168(12):1397-1408.
2. Michelozzi P, Accetta G, De Sario M, D’Ippoliti D, Marino C, et al (2009) High temperature and hospitalizations for cardiovascular and respiratory causes in 12 European cities. Am J Respir Crit Care Med 179(5):383-389.
3. Kovats RS, Hajat S, Wilkinson P (2004) Contrasting patterns of mortality and hospital admissions during heatwaves in London, UK. Occup Environ Med 61:893-898.
