# Supplementary material for: Apparent Temperature and Cause-Specific Emergency Hospital Admissions in Greater Copenhagen, Denmark
Source: PLoS One. 2011 Jul 29;6(7):e22904. doi: 10.1371/journal.pone.0022904 (PMC3146500; doi:10.1371/journal.pone.0022904)
Supplement: Text S4 — Sensitivity analyses: alternative temperature definition. (DOC) [file pone.0022904.s021.doc]

**Text S4**

**Sensitivity analyses: alternative temperature definition**

Barnett and colleagues concluded that there is no single temperature measure that is superior to others [1]. The robustness of the observed Tapp_max_ associations (Tables 3 and 4, Figure S1) was confirmed in models with the alternative temperature definition: 24-hr average temperature (T_ave_). Figure S8 illustrates the % change in the cause-specific admissions per IQR increase in the different lags of T_ave_ during the warm and cold periods, respectively, after adjusting for 24-hr mean relative humidity (RH_ave_), public holidays and weekly influenza rates, but not for any of the pollutants. A similar lag structure was observed as in the Tapp_max_ models (Figure S1).

Figure S9 illustrates the % change in the cause-specific admissions per IQR increase in the different lags of T_ave_ during the warm and cold periods, respectively, after adjusting for public holidays and weekly influenza rates, but not for RH_ave_ nor for any of the pollutants. A similar lag structure was observed as in the Tapp_max_ models (Figure S1).

Similar effect estimates were observed in the warm period for the CA5 of Tapp_max_ and T_ave_, after adjusting for the CA5 of RH_ave_, public holidays, weekly influenza rates and the CA5 of PM_10_ (CVD hospital admissions) and the CA5 of NO_2max_ (CBD hospital admissions)(Compare Table 3 and Table S4). The effect estimates of the CA5 of T_ave_ in the RD hospital admission models differ substantially, when adjusting for both the CA5 of RH_ave_ and PM_10_, compared to those of T_ave_, without adjusting for RH_ave_ (Compare Tables S4 and S6) or those of Tapp_max_ (Table 3).

Table S8 reports the Spearman correlation coefficients between T_ave_, RH_ave_ and the pollutants. Similar correlations were observed between Tapp_max_, T_ave_ and the pollutants (Compare Table 2 and Table S8). In the warm period RH_ave_ had a negative and positive correlation with T_ave_ and PM_10_, respectively, whilst the correlations were both positive in the cold period. The correlation between RH_ave_ and PM_10_ was stronger in the warm period. RH_ave_ was not significantly associated with any of the outcomes (results not shown), except with RD hospital admissions in the warm period (inverse association). This may explain the different results observed for RD hospital admissions in the T_ave_ + RH_ave_ + PM_10_ model, T_ave_ + PM_10_ model and Tapp_max_ + PM_10_ model. Other studies did not highlight or investigated such an anomaly. We observed similar results for RD hospital admissions in the T_ave_ + RH_ave_ model, T_ave_ model and Tapp_max_ model.

The exclusion of RH_ave_ in the T_ave_ models gave similar effect estimates in the warm period for the CA5 of T_ave_ compared to the Tapp_max_ models, after adjusting for the CA5 of PM_10_ (CVD hospital admissions), CA5 of NO_2max_ (CBD hospital admissions), public holidays and weekly influenza rates (Compare Table 3 and Table S6).

The T_ave_ models with or without RH_ave_ gave similar effect estimates in the cold period for the CA5 of T_ave_ compared to the Tapp_max_ models, after adjusting for public holidays, weekly influenza rates (CVD and CBD hospital admissions) and the CA5 of PM_10_ (RD hospital admissions)(Compare Table 4, Tables S5 and S7).

**References**

1. Barnett AG, Tong S, Clements ACA (2010) What measure of temperature is the best predictor of mortality? Environ Res 110: 604-611.
